# Understanding Ferguson's *δ*: time to say good-bye?

**DOI:** 10.1186/1477-7525-7-38

**Published:** 2009-04-30

**Authors:** Berend Terluin, Dirk L Knol, Caroline B Terwee, Henrica CW de Vet

**Affiliations:** 1 Department of General Practice and the EMGO Institute for Health and Care Research, VU University Medical Centre, Amsterdam, the Netherlands; 2 Department of Clinical Epidemiology and Biostatistics, and the EMGO Institute for Health and Care Research, VU University Medical Centre, Amsterdam, the Netherlands

## Abstract

A critique of Hankins, M: 'How discriminating are discriminative instruments?' *Health and Quality of Life Outcomes *2008, 6:36.

## Background

Recently Hankins (re-)introduced Ferguson's coefficient *δ *as an index of discrimination, to be distinguished from the well-known measurement properties validity and reliability [1,2]. Hankins presented Ferguson's *δ *as a useful index of the degree to which an instrument discriminates between individuals, being "the ratio of the observed number of between-person differences to the theoretical maximum number possible" [1]. The value of *δ *varies between 0 (no discrimination at all) and 1 (maximal possible discrimination). The calculation is straightforward and Hankins provided a generalized formula for calculating *δ *for questionnaires with dichotomous as well as polytomous items.

Hankins' paper [1] elicited two critical comments [3,4]. Wyrwich referred to the work of Guyatt [5] who related discrimination tot reliability, theoretically consistent correlations with other measures, and interpretability of small but important differences. Since Hankins failed to present relevant information regarding these issues, Wyrwich concluded that it is impossible to make a judgement on whether Ferguson's *δ *is a useful index or not [3]. Whereas Hankins stated that discrimination is something else than reliability, Norman expressed the opposite view, i.e. that "reliability *is *discrimination". Scrutinizing Hankins' examples and adding one of his own, Norman illustrated his main point that Ferguson's *δ *fails to distinguish between true differences and measurement error [4]. In his response, Hankins remarked that both Norman and Wyrwich made too much of his examples, and seemed to have missed his point, which is that Ferguson's *δ *is an additional index of an instruments' measurement properties, beside reliability, validity and interpretability, and that Ferguson's *δ *can only be computed on the assumption that the measurement is valid and reliable [6].

In this letter, we will examine how exactly Ferguson's *δ *'works' and what *δ *actually measures. More specifically, we will show that the magnitude of *δ *is only determined by the distribution of the scores in a given sample. Moreover, we will show that the standard computation of *δ *ignores reliability, but, when reliability is accounted for, *δ *becomes impossible to interpret. Our final conclusion will be that Ferguson's *δ *is not a useful attribute of a measurement instrument.

## How Ferguson's *δ *works

The formula of *δ*, presented by Hankins, reads:

(1)

in which *k *is the number of items, *m *is the number of response options per item, *n *is the sample size and  is the sum of squared frequencies of each score *i*. Note that *k*(*m *- 1) equals the score range of a scale, and 1 + *k*(*m *- 1) equals the total number of score categories *q *of an instrument.

### Example 1

**Figure 1:**
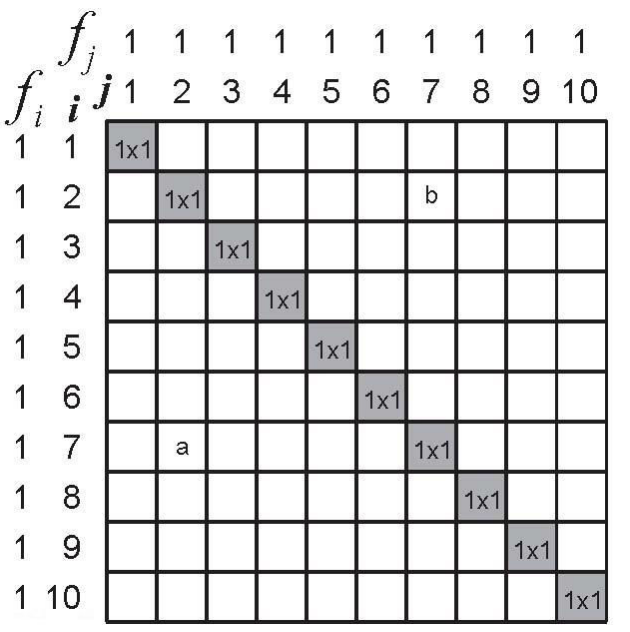
**Graphical representation of how Ferguson's *δ *'works'**. Ferguson's *δ *counts comparisons between subjects. In this sample 10 subjects are mutually compared. The subjects have scores 1, 2, ... 9, 10 on an instrument with 10 score categories (*i*). The frequency (*f*_*i*_) is 1 for all *i *scores. The subjects are placed in a 10 × 10 matrix in which each cell comprises 1 comparison of 1 subject with another subject (white cells) or with itself (shaded cells). Ferguson's *δ *relates the number of discriminating comparisons between subjects (white cells) to all comparisons (all cells). See the text for the actual calculation of *δ*.

In order to illustrate how Ferguson's *δ *'works', let us consider a situation in which 10 subjects have each obtained a unique score on some instrument between 1 and 10. Thus, the subjects' scores are 1, 2, ..., 9, 10. In addition, let us assume that the scale is perfectly reliable (reliability coefficient: 1), so that the scores represent 'true' scores. The distribution of the scores is uniform: the *n *= 10 subjects are evenly distributed over the *q *= 10 score categories. Since all *q *= 10 possible scores have a frequency of 1, Ferguson's *δ *is:



Intuitively, it may already have been apparent that this example presents a maximally discriminative instrument: each subject is perfectly distinguished from all other subjects. Therefore, it comes as no surprise that *δ *is 1 (the maximum value). Figure 1 illustrates how *δ *is calculated: in a matrix *n *subjects (rows) are compared with the same *n *subjects (columns). In every cell of the matrix, one subject (from the rows) is compared to one subject (from the columns). Ferguson's *δ *classifies these comparisons as either the same (when *i *= *j*) or as different (when *i *≠ *j*). In formula (1) we see *n*^2 ^in the denominator: all possible (*n *× *n*) comparisons between the *n *subjects, all cells in Figure 1. In the numerator we see the expression , the sum of comparisons of each subject with his or her self: the shaded cells in Figure 1. The expression  represents the between-subjects comparisons of different subjects: the white cells in Figure 1. If we re-write the formula of *δ *as

(2)

it is easy to see that *δ *contains the ratio between all discriminating comparisons (the white cells in Figure 1) and all possible comparisons (all cells in Figure 1, white and shaded). In addition, the formula contains a correction for the number of score categories *q*. When we re-write the formula as



it becomes apparent that the denominator is corrected for the fact that a person cannot be discriminated from his/her self (the shaded cells). Instead of all possible *n*^2 ^comparisons (all cells), the denominator represents all possible *discriminating *comparisons (the white cells). Note that all discriminating comparisons are counted twice. For instance, the subject with score '7' is compared with the subject with score '2' in two cells (see Figure 1): cell a contains the comparison between subject (*i *= 7) and subject (*j *= 2), while cell b contains the comparison between subject (*i *= 2) and subject (*j *= 7), and it should be remembered that subject (*i *= 2) and subject (*j *= 2) are the same, and the same goes for subject (*i *= 7) and subject (*j *= 7). It should also be noted that Ferguson's *δ *treats the score categories as the scores of a nominal (or categorical) scale: all differences (if present) between all subjects are valued equally. In case the scale has ordinal properties (as in Hankins' examples) Ferguson's *δ *does not utilize the variation in differences between subjects.

### Example 2

**Figure 2:**
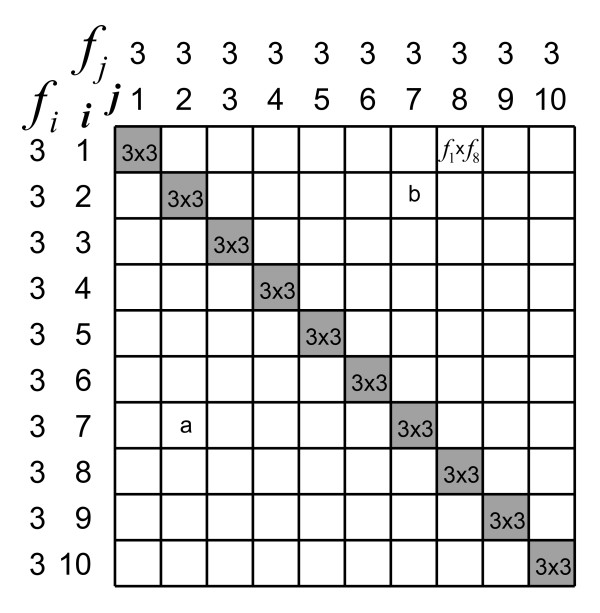
**Graphical representation of how Ferguson's *δ *'works'**. In this sample 30 subjects are uniformly distributed over a scale with 10 score categories (*i*), so that the frequency (*f*_*i*_) for all *i *scores is 3. The subjects are placed in a 10 × 10 matrix according to their scores. Each cell comprises *f*_*i *_× *f*_*j *_comparisons of *f*_*i *_= 3 subjects with a certain score (e.g. '7') with *f*_*j *_= 3 subjects with another score (e.g. '2') in the white cells, or with themselves in the shaded cells. Ferguson's *δ *relates the number of discriminating comparisons between subjects (within the white cells) to all comparisons (within all cells). See the text for the actual calculation of *δ*.



Now, let us calculate *δ *for a situation in which, again, *q *= 10, but we have a larger sample size, *n *= 30. Again, the subjects are uniformly distributed over the 10 score categories and we assume no measurement errors (Figure 2). Ferguson's *δ*, using formula (2), is now:

Note that the cells in Figures 2 contain numbers of comparisons between subjects, e.g. cell a contains 9 comparisons between 3 subjects with scores '2' and 3 other subjects with scores '7' (note that cell b contains the same comparisons). Ferguson's *δ *counts comparisons between subjects *within score categories*. The shaded cells comprise comparisons among subjects with the same scores (*i *= *j*), whereas the white cells comprise comparisons between subjects with different scores (*i *≠ *j*). That Ferguson's *δ *is independent of the sample size *n *can be derived from the formula of *δ*. If *p*_*i *_is the proportion of subjects within score category *i *, then Ferguson's *δ *becomes:



Furthermore, it can be shown that, under the assumption of a uniform distribution, *δ *is always 1, irrespective of the number of score categories *q*. In a uniform distribution, all score categories comprise the same proportion of subjects, namely . Therefore, Ferguson's *δ *becomes:



So, Ferguson's *δ *is always 1, irrespective of the number of score categories *q*, provided that the subjects are evenly (uniformly) distributed among the score categories. Even in the case of *q *= 2 Ferguson's *δ *remains 1 as long as half of the subjects score '1' and the other half of them score '2'. Whether this situation represents an example of excellent discrimination, seems to be questionable. Intuitively, one expects an instrument to lose discriminative power when the number of score categories is limited to very small numbers, i.e. 2 or 3.

## Reliability

### Example 3

**Figure 3:**
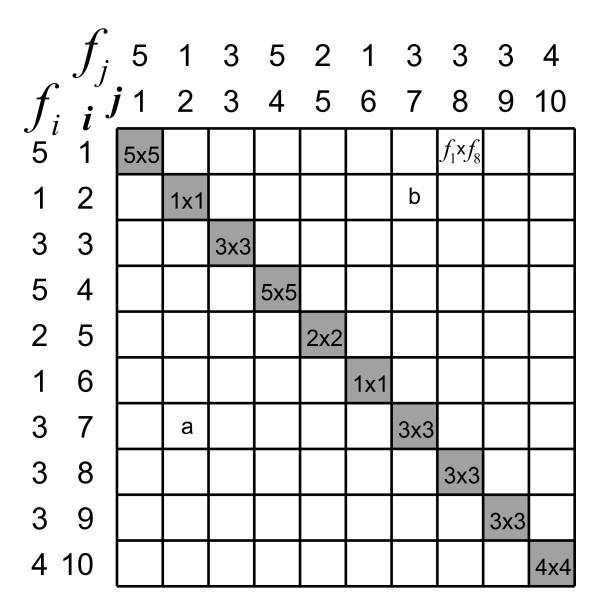
**The impact of measurement error on Ferguson's *δ***. Graphical representation of the same 30 subjects, and their mutual comparisons, as in Figure 2, but now with a little measurement error added to their scores, resulting in different frequencies (*f*_*i*_) per score category.



**Figure 4:**
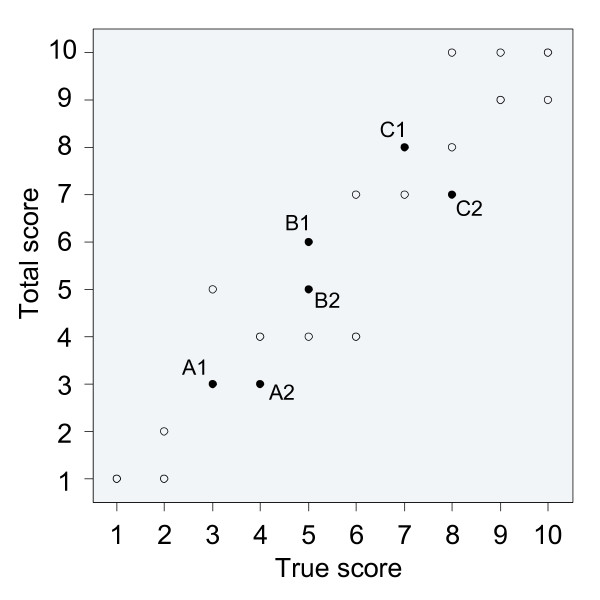
**The impact of measurement error on discrimination and ordering**. Scatterplot comparing the total scores (true score plus measurement error) of the 30 subjects of Figure 3, with their true scores. Three pairs of subjects have been highlighted to illustrate changes in discrimination and ordering due to measurement error.

So far, we assumed an instrument without measurement error, an unrealistic situation. What will happen with Ferguson's *δ *when we introduce some error into the scores? We will continue with the sample of Example 2, and assume the scale is ordinal. In Example 3, however, we add some measurement error. In order to obtain scores between 1 and 10 with a 'good' reliability coefficient between 0.80 and 0.90, we add to the perfectly reliable (true) score of the subjects a normally distributed random (error) score with a mean of 0 and a standard deviation of 1. After summating the true score and the error score, we need to 'force' the scores into the score categories by rounding to the nearest integer and subsequently recode scores <1 into 1 and scores >10 into 10. The resulting total score turns out to have a variance of 9.91. The variance of the true score is 8.53, and the variance of the error score thus is 9.91 – 8.53 = 1.38. Hence, the reliability coefficient of the score is 8.53/9.91 = 0.86. The situation is shown in Figure 3. The number of non-different comparisons,  (within the shaded cells), is 108. Using formula (2), Ferguson's *δ *is:

This example suggests that Ferguson's *δ *is hardly affected by measurement error. However, if we compare the total scores (true + error) with the true scores (Figure 4), we see that the discrimination between subjects arises from error in many cases. For illustrative purposes, three pairs of subjects in Figure 4 have been highlighted. Subjects A1 and A2, who are truly different, end up in the same score category, so they cannot be discriminated, due to error. On the other hand, subjects B1 and B2, who have the same true score, end up being discriminated from each other, due to error. For subjects C1 and C2 the very ordering of their discrimination has been reversed: C2 scores higher than C1 on the true score, but C1 scores higher on the total score due to measurement error. All these erroneous discriminations do not seem to affect *δ *at all.

This example illustrates what Norman already advanced, namely that Ferguson's *δ *does not distinguish between true differences and differences due to measurement error [4]. In his words: "The problem with *δ *is that all it cares about are differences". Hankins replied that 'acceptable' reliability (and validity) must be presupposed in order to determine *δ*. Furthermore, Hankins suggested that the computation of *δ *should be adjusted for non-reliable differences, to take into account only meaningful differences [6]. By current standards, the reliability of the scale in our example is fully 'acceptable' (reliability coefficient 0.86). Let us execute the suggested adjustment of *δ *for reliability, by assuming that the 'smallest detectable difference' (SDD) [7] is a meaningful difference between subjects. The SDD is the smallest difference between two subjects that can, with 95% confidence, be attributed to a real difference in true scores. The SDD can be calculated from the standard error of measurement (SEM) using the formula .

**Figure 5:**
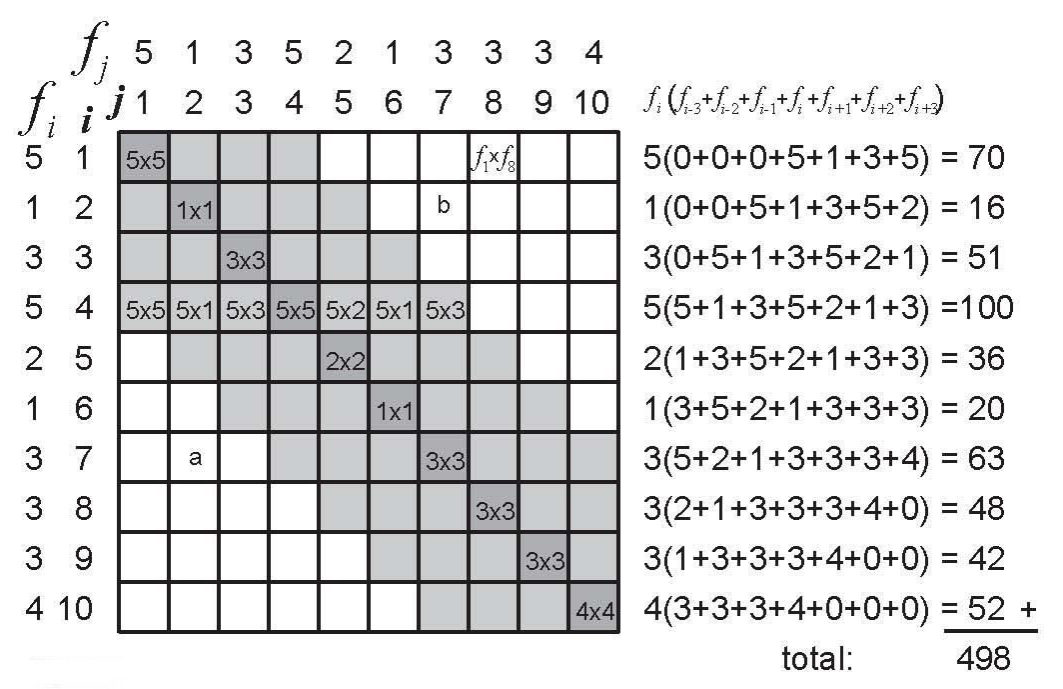
**Adjusting Ferguson's *δ *for non-reliable differences**. Elaboration of Figure 3 to illustrate how Ferguson's *δ *can be adjusted for non-reliable differences. The lightly shaded cells comprise comparisons of subjects whose differences fall below the smallest detectable difference, which is 3.24 in this case.



The SEM is the square root of the error variance: . That makes SDD = 3.24. So, differences between subjects ≤ 3 must be included in the -term in the numerator in formula (2). The formula of *δ *now becomes as follows:



in which by definition *f*_*j *_= 0 when *j *< 1 or *j *> *q*. Figure 5 illustrates the calculation. Cells in which between-subject differences are 3 or smaller are lightly shaded. Ferguson's *δ*, adjusted for non-reliable differences, can be calculated for our Example 3 as:

This result suggests that adjusting *δ *for non-reliable differences might have a large impact on its magnitude, even when reliability is 'acceptable'. But, what does that tell us about the discriminative power of this instrument? What represents *δ *after adjustment for non-reliable differences? We really don't know.

## Distribution

Hankins reported that Ferguson mentioned that *δ *was 1 when the distribution was uniform (as we confirmed), and that normal distributions typically produce *δ *values of about 0.90 [1]. Lower values of *δ *are associated with skewed distributions.

In daily life, uniform distributions are highly uncommon. More common are normal and skewed distributions. In addition, many health outcomes are characterized by floor or ceiling effects. We will now examine how *δ *is affected by different kinds of distributions.

### Example 4

**Figure 6:**
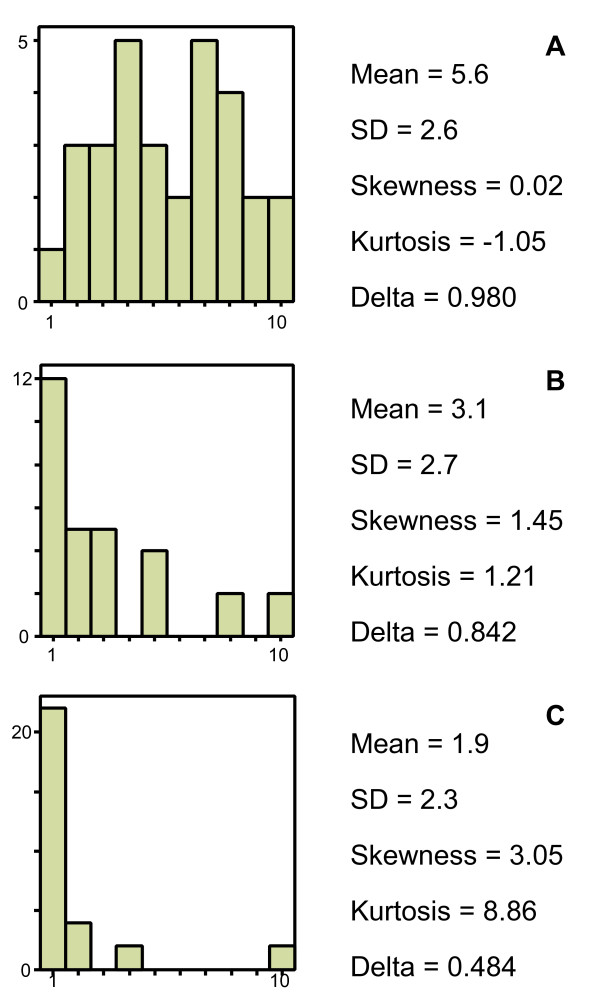
**The impact of distribution on Ferguson's *δ***. Illustration of the association between the score distribution and Ferguson's *δ *using simulated data. Figure A represents a normal distribution (*n *= 30); Figure B represents a skewed distribution (*n *= 30); Figure C represents a highly skewed distribution with a marked 'floor effect' (*n *= 30).

Consider 30 subjects displaying a normal distribution on a 10-point scale (Figure 6a).  is 106. The standard computation of Ferguson's *δ *yields: .

Now, let us examine what happens to *δ *when the distribution is skewed. A skewed distribution is often present in health outcomes when the majority of subjects are normal, healthy or well. We construct a skewed distribution by taking the fourth power of the scores of the normal distribution, adjusting the range to the 1–10 range and rounding the scores to the nearest integer (Figure 6b).  is 218. Ferguson's *δ *is 0.842.

A more skewed distribution is made by taking the tenth power of the scores of the normal distribution, adjusting the range to the 1–10 range and rounding the scores to the nearest integer (Figure 6c).  is 508. Ferguson's *δ *is 0.484.

In the skewed distributions there is a clear floor effect discernable. These examples and some others we have tried, suggest that a decrease of *δ *is associated with kurtosis, the clustering of subjects within one or a few response categories. If *δ *is indeed a reflection of the sample's distribution, it does not seem to tell us anything about the discriminative properties of the instrument.

### Example 5

**Figure 7:**
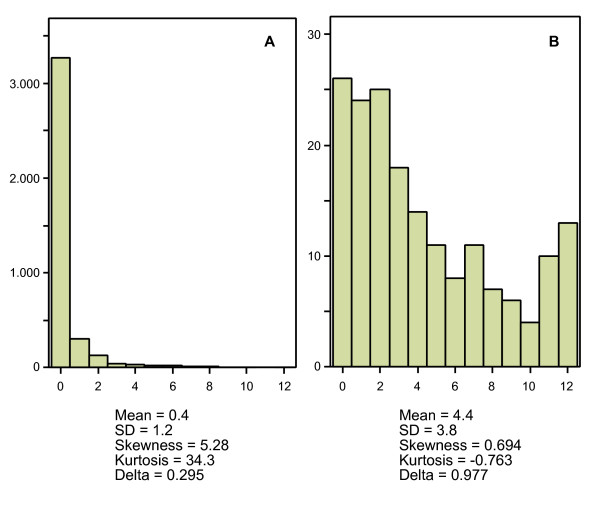
**Same scale, different *δ *values**. Illustration of the association between the score distribution and Ferguson's *δ *using real life data. Figure A represents the distribution of the 4DSQ depression scale in a sample of employees (*n *= 3852); Figure B represents the distribution of the same depression scale in a sample of general practice patients with depressive symptoms (*n *= 177).

Finally, we will present a real life example of a single instrument in different populations. The instrument is the depression scale of the Four-Dimensional Symptom Questionnaire (4DSQ), which has 6 items, each with 3 response options [8]. So, the total number of score categories of the scale *q *is 13. In a sample of employees (*n *= 3852) Cronbach's *α *was 0.82 [9]. We found that 84,8% of the employees scored '0' (Figure 7a). In this case *δ *turned out to be as low as 0.295. In another sample of general practice patients with depressive symptoms (*n *= 177) Cronbach's *α *was 0.90 [10]. In this sample only 14.7% of the subjects scored '0' (Figure 7b). In this case *δ *turned out to be as high as 0.977. The same instrument, with the same reliability and validity, produced highly different *δ *values in different populations, due to differences in distributions. Again, this has nothing to do with the discrimination of the instrument.

## Discussion

We have shown that Ferguson's *δ *is only determined by the distribution of the subjects in a sample over de score categories of an instrument. If the distribution is uniform, then *δ *is always 1. To our surprise, the maximum value of *δ *turned out not to be limited by the number of response categories *q*. Because, at any given value of *q *(provided *q *> 1), *δ *can take on any value between 0 and 1, it is safe to say that *δ *is independent of *q*, the number of score categories of the instrument.

Does Ferguson's *δ *say anything about the discriminative power of an instrument? Take for example our real life example. Is it valid to say that the 4DSQ depression scale is poorly discriminative in an employee sample, just because it fails to discriminate among those employees who do not experience the kind of depressive symptoms that de scale measures? If we want to discriminate anything with the 4DSQ depression scale, then we want to discriminate those who do experience depressive symptoms from those who don't, and that is what the scale is doing reasonably well [8]. There seems to be absolutely no point in requiring that a depression scale discriminates among individuals who do not have depressive symptoms. To put it in more general terms, there is no point in discriminating among people who belong to the same category.

We agree with Norman's point [4] that Ferguson's *δ *simply ignores measurement error. Ferguson's *δ *does not distinguish between reliable and non-reliable differences. Although it is technically possible to adjust *δ *for non-reliable differences, this has a large impact on its magnitude. More problematic, though, is that we don't know how the resulting statistic should be interpreted. The important point is, that in the standard computation of *δ *reliability is not an issue. Hankins provided an example of an 8-item scale with a reliability coefficient (Cronbach's *α*) of 0.76 and a *δ *of 0.92 [1]. Surely, this *δ *had not been adjusted for non-reliable differences!

## Conclusion

The conclusion seems inescapable that Ferguson's *δ *is a characteristic of a population and that it does not refer to any useful property of a measurement instrument. We therefore conclude that it is time to say good bye to Ferguson's *δ *and let it slip into oblivion again.

## Competing interests

The authors declare that they have no competing interests.

## Authors' contributions

HdV and BT conceived of the idea for the paper. BT and DK worked out the statistical issues. BT drafted the manuscript. All authors contributed to discussions and critical comments on previous versions of the manuscript, and read and approved the final version.
